# Daily dataset of oil prices and stock prices for the top oil exporting and importing countries from the region of Asia

**DOI:** 10.1016/j.dib.2019.104871

**Published:** 2019-11-23

**Authors:** Saleha Ashfaq, Rashid Maqbool, Yahya Rashid

**Affiliations:** a School of Economics and Management, Fuzhou University, Fujian, P.R. China; b CENTRUM Católica Graduate Business School (CCGBS), Lima, Peru; c Pontificia Universidad Católica del Perú (PUCP), Lima, Peru; d Mechanical Engineering Department, Prince Sattam Bin Abdulaziz University, Al-Kharj, Kingdom of Saudi Arabia

**Keywords:** Oil prices, Stock market, Oil-export, Oil-import

## Abstract

This data presented in this article is specifically employed from the Asian region based on the top position in the list of oil exporting and oil-importing countries around the world. Asia as the biggest continent on the earth had high consumption of energy [1]. Here we employed the daily prices of crude oil and seven oil trading countries, out of which three are oil exporting (Saudi Arabia, United Arab Emirates, Iraq) and four are oil-importing countries (China, Japan, South Korea, India), from the time period of 1-09-2009 to 31-08-2018. The data is collected from an authentic database Bloomberg. This data is related to the research paper “Volatility spillover impact of world oil prices on leading Asian energy exporting and importing economies' stock returns. Energy, 188 (2019), 116002, https://doi.org/10.1016/j.energy.2019.116002 [2]”. This data is useful to compare the oil prices impact on the leading oil trading countries and also compare a set of countries affected most by oil prices’ fluctuations, oil-exporting countries or oil-importing countries. Since this data covers the period of latest oil-crisis, so the impact of oil-crisis could also be analysed.

**Table:** Specifications Table

Subject	Energy
Specific subject area	Oil prices impact on Asian Oil-exporting and Oil-importing countries
Type of data	Table Graph Figure
How data were acquired	The daily stock prices are acquired from Bloomberg. Oil prices data is acquired from the official website of Energy Information Administration (EIA).
Data format	Raw
Parameters for data collection	Countries of the data was selected on the bases of their top 5 ranking in the list of oil-exporting and oil-importing countries.
Description of data collection	The data of daily stock prices is employed for the period from 01 to 09-2009 to 31-08-2018. WTI crude oil prices were taken for the oil prices, and overall data was collected from the official website of Energy Information Administration (EIA). Seven Asian countries were chosen from the list of top Asian oil trading countries, out of which three were oil-exporting countries (Saudi Arabia, United Arab Emirates, Iraq) and four were oil-importing (China, Japan, South Korea, India) countries' stocks. Data was collected from an authentic database Blomberg.
Data source location	Bloomberg (an online data source)
Data accessibility	Data is available in the supplementary file attached with this article.
Related research article	Ashfaq, S., Tang, Y., & Maqbool, R. (2019). Volatility spillover impact of world oil prices on leading Asian energy exporting and importing economies' stock returns. *Energy, 188* (2019), 116002. https://doi.org/10.1016/j.energy.2019.116002

**Table:** 

**Value of the Data** • Following points highlight the importance of the data. Please see these points; • This data is useful for analysing the oil price impact on Asian top oil trading ae well as hedging and portfolio management. • This data can be beneficial for hedger and policy maker who are interested in Asian region. • The data can be further used for more comparison with further Asian countries as well as with other regions of the world. • The data also covers the impact of most recent oil-crisis on the leading oil-trading countries' stock markets.

## 1 Data

Energy is the key trade commodity in today’s world, in which the Asian region is predominated with its huge energy consumption level [[1], [2]]. Energy is the backbone for any economy [[3], [4], [5]], and oil is considered to be a major source of energy [2], used in daily life of mankind as a key part of its environmental components such as; industry, transportation and infrastructure etc [6]. This data is comprised of oil price and Asian leading oil trading countries. Here the data of daily stock prices is employed for the period from 01 to 09-2009 to 31-08-2018. However, for the oil price WTI crude oil prices are selected and data was collected from the official website of Energy Information Administration (EIA). Seven Asian countries were chosen from the list of top Asian oil trading countries, out of which three were oil-exporting countries (Saudi Arabia, United Arab Emirates, Iraq) and four were oil-importing (China, Japan, South Korea, India) countries' stocks. Data was collected from an authentic database Blomberg. The selection of this data is based on these countries’ top positioning in the list of world ranking for oil-exporting and oil-importing countries. This ranking is based on the information provided by the “BP Statistical Review of World Energy, 67th Edition” and “The World Facebook” a source of “Central Intelligence Agency”. According to which the selected Asian countries lies in the list of top five oil-exporting and oil-importing countries. Moreover, this data is really attractive for the hedging, speculation and portfolio managment purpose, as well as for those policy makers who are intrested in the Asian region. The time duration selected for this dataset covers the impacts of latest big oil crises periods such as; post-crises impact of oil crisis (2003–2009) and the affacts of recent oil crisis (2014–2016). Furthermore, this data also elaborate the pre and post impacts of oil crises on the most important markets of oil trading countries of Asia.

## 2 Experimental design, materials, and methods

The data covers the daily stock prices of three oil-exporting and four oil-importing countries, and then return of these prices are employed. The returns are calculated by taking the natural logarithm of closing prices divided by lagged closing prices.

$$R_{i,t}=\ln\left(\frac{P_{i,t}}{P_{i,t-1}}\right)\times 100,\;i=stock,\;oil$$

**Fig. 1:**
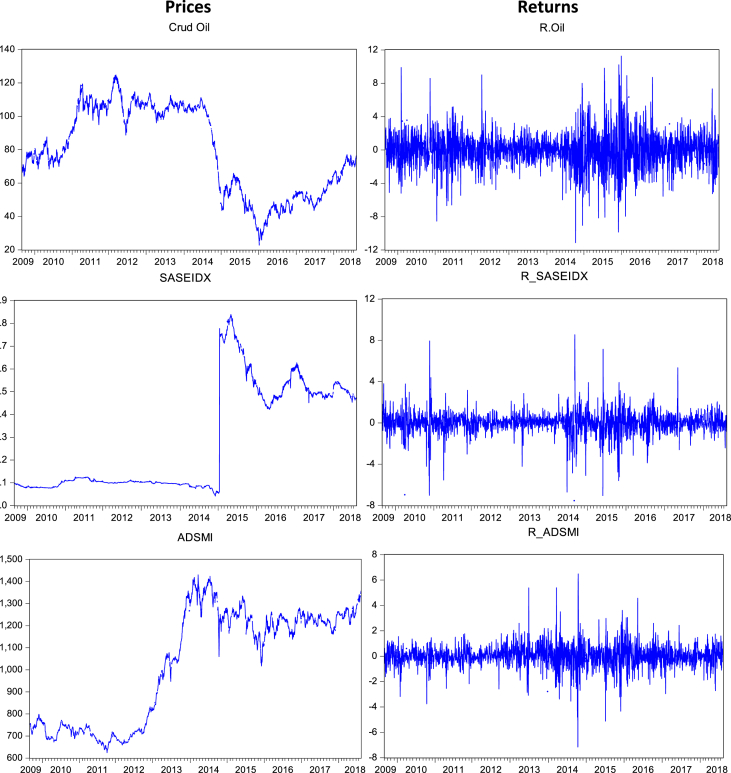

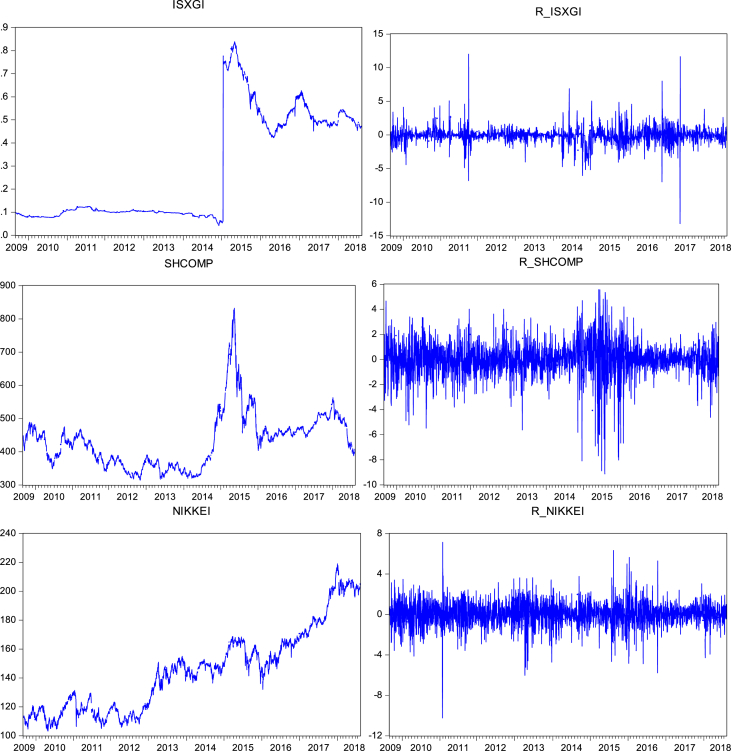

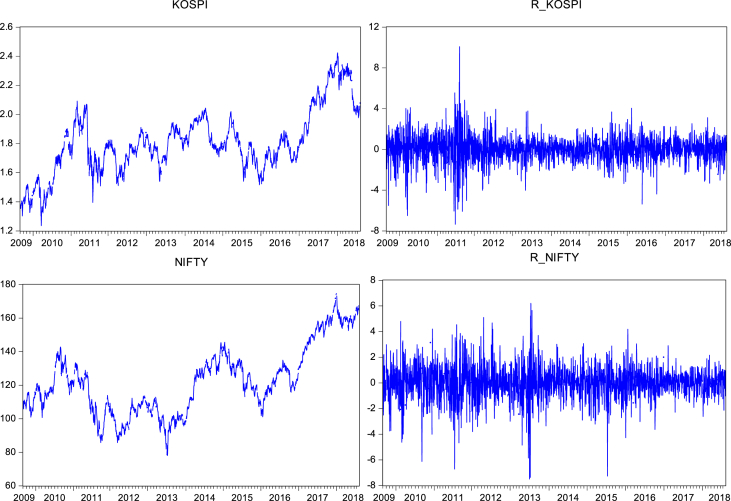
Graphs of prices and returns.

At first the graphical pictures of prices and returns are presented (Fig. 1) to analyze the trend of the prices and return movements in the selected time zone. In these graphs (Fig. 1) we can also easily analyze the sudden upward or downward movement of the prices and returns line, which show the effects of recent big oil-crisis as well as pre and post crisis effects on the stock markets of top oil-trading countries of Asia. Further, we also run a basic descriptive analysis (Table 1), as the variables are originated from the different state of the economy and converted into USD, it exhibits the mixed statistic results as from positive to negative.

**Table 1:** Descriptive Analyses Table.

	R_OIL	R_SASEIDX	R_ADSMI	R_ISXGI	R_SHCOMP	R_NKY	R_KOSPI	R_NIFTY
Mean	−0.007009	0.016229	0.027174	−0.071621	−0.003550	0.028307	0.019930	0.019351
Median	0.062105	0.077716	0.034274	−0.054152	0.053709	0.033921	0.077510	0.068818
Maximum	11.28922	8.547398	6.487471	12.00381	5.572456	7.130135	10.07229	6.201057
Minimum	−11.12576	−7.557433	−7.154925	−13.19741	−9.149866	−10.23041	−7.367754	−7.514299
Std. Dev.	2.102116	1.078309	0.868071	1.198000	1.420537	1.259193	1.294144	1.274191
Skewness	0.113221	−0.618433	−0.162456	0.174760	−0.967097	−0.416673	−0.217798	−0.349534
Kurtosis	6.181133	14.17175	11.08619	22.85729	8.817929	7.332427	7.574103	6.445702
Jarque-Bera	914.9556	11365.14	5891.557	35482.64	3381.480	1750.984	1899.215	1112.025
Probability	0.000000	0.000000	0.000000	0.000000	0.000000	0.000000	0.000000	0.000000
Sum	−15.13175	35.03899	58.66830	−154.6307	−7.665403	61.11422	43.02784	41.77926
Sum Sq. Dev.	9535.967	2509.215	1626.155	3097.171	4354.686	3421.653	3614.239	3503.648
Observations	2159	2159	2159	2159	2159	2159	2159	2159

Descriptive analysis was performed to present a real picture about the important trends of the oil trading countries (both type of countries, including the top oil-exporting and oil-importing countries). The details of the descriptive analysis for the data are presented in Table 1.

## Ethics approval

Ethics approval is not applicable.

## Consent for publication

The authors of the study have given their consent for the data to be used and published in this scientific article.

## Availability of data and materials

Data generated or analysed during the study are available in the published paper which is highlighted in the aforementioned text. Moreover, raw data is linked with this data article in the supplementary file.

## Funding

There are two kind of funding supports with this data article.

(1)- Financial support from Fujian Major Social Science Planning Fund Program under grant no. FJ2017Z006.
(2)- Fujian Natural Science Foundation Project under grant no. 2017J01518.

## Authors' contributions

All of the authors have contributed equally to the design of theoretical model, to analyzing and discussing the data and to writing the data article. All of the authors read and approved this data article.

## References

[1] Sarwar S., Khalfaoui R., Waheed R., Dastgerdi H.G. (2019). Volatility spillovers and hedging: evidence from Asian oil-importing countries. Resour. Policy.

[2] Ashfaq S., Tang Y., Maqbool R. (2019). Volatility spillover impact of world oil prices on leading Asian energy exporting and importing economies' stock returns. Energy.

[3] Maqbool R. (2018). Efficiency and effectiveness of factors affecting renewable energy projects; an empirical perspective. Energy.

[4] Maqbool R., Sudong Y. (2018). Critical success factors for renewable energy projects; empirical evidence from Pakistan. J. Clean. Prod..

[5] Maqbool R., Rashid Y., Sultana S., Sudong Y. (2018). Identifying the critical success factors and their relevant aspects for renewable energy projects; an empirical perspective. J. Civ. Eng. Manag..

[6] Wu X., Gao M., Guo S., Maqbool R. (2019). Environmental and economic effects of sulfur dioxide emissions trading pilot scheme in China: a quasi-experiment. Energy Environ..

